# Engineering Biology of Yeast for Advanced Biomanufacturing

**DOI:** 10.3390/bioengineering10010010

**Published:** 2022-12-21

**Authors:** Wei Jiang, Yanjun Li, Huadong Peng

**Affiliations:** 1 The Novo Nordisk Foundation Center for Biosustainability, Technical University of Denmark, 2800 Kongens Lyngby, Denmark; 2 College of Biotechnology, Tianjin University of Science and Technology, Tianjin 300457, China

Advanced biomanufacturing has been widely involved in people’s daily life, such as the production of molecules used as pharmaceuticals, in foods and beverages, and in bio-fuels [1]. Yeast is a micro-organism that has been used by humans for thousands of years for the production of food and beverages. Its versatility and robustness under fermentation conditions, along with its generally recognized as safe (GRAS) status and ease of culture, have made it a valuable tool in biotechnology [2,3]. Yeast *Saccharomyces cerevisiae* is the best-studied and most widely used yeast species in industrial applications [4]. However, other species, such as *Pichia pastoris*, *Hansenula polymorpha*, *Yarrowia lipolytica*, and *Kluyveromyces marxianus*, are also emerging as valuable tools in engineering biology. These species have unique characteristics that make them suitable for specific applications, such as the ability to grow at low temperatures or the production of certain compounds [5,6]. Overall, the diversity of yeast species and their ability to be genetically modified make them valuable tools in advanced biomanufacturing.

To provide a forum for scientific dissemination, the Special Issue, ‘From Yeast to Biotechnology’, compiles the most relevant papers in yeast biotechnology, which not only provide the latest progress of yeast in biotechnology but also gives readers an excellent insight into this field.

Yeast has been the crucial model organism for both academia and industry for more than 40 years. With the exploitation of increasingly novel strategies and skills, yeast-based biotechnology is constantly boosted. The development of genetic engineering achieved the writing of DNA in yeast *S. cerevisiae*. The Sc2.0 project is drawing to a close, providing a precious reference for synthetic genomics. This also opens new horizons to tackle many challenges of humankind and breaks the limits of science [7]. In addition, omics analysis has become a powerful technology to characterize and understand the metabolic network in yeast. Based on omics analysis, some complex biological systems can be better manipulated to establish robust biomanufacturing platforms [8]. For example, the ability of *S. cerevisiae* to produce ethanol and utilize the fermented carbon source plays a key role in the industries of brewing, winemaking, and ethanol production. The proteomic study of *S. cerevisiae* related to different fermentation processes revealed the differences in protein expression levels between processes, which shows that different typical genes respond to different technological environments. These data can be further applied to strengthen the industrial yeast strain to adapt to different biotechnological environments [9]. Interestingly, optogenetics is also introduced to dynamically control yeast cellular processes in recent years, aiming to improve poor productivity. The metabolic flux is directed toward production through establishing the optogenetic systems in yeast. This new system brings in a new field of vision for optimizing yeast-based microbial cell factories [10]. 

Gene regulatory network modeling is another vital tool to improve the bioproduction ability of yeast. Triacylglyceride (TAGs) biosynthesis-related genes were classified in the oleaginous yeast *Lipomyces starkeyi,* which was subsequently used to construct the different gene regulatory network modeling. The modeling was used to analyze the oil- and TAG-related regulatory factors that could be further engineered in *Lipomyces starkeyi* to improve its lipids production ability [11]. Yeast is also an ideal model micro-organism to investigate various molecular mechanisms that are applicable from yeast to mammals. To reveal how mammals cope with copper as an essential metal element for life, yeast *S. cerevisiae* was used as a model to study this mechanism. It is found that *S. cerevisiae* cells could form deposits on the cell wall to tolerate high copper concentration, especially the copper-containing deposits accumulated on media, with reducing sugars as the sole carbon source rather than nonreducing carbon sources. The vigorous agitation could prevent copper-containing deposits at the cell wall. The copper deposits on the cell wall could be increased by disrupting the low-affinity copper intake through the plasma membrane. Based on this mechanism, we could propose a helpful approach for removing excess copper from different contaminated solids [12].

In addition to exploiting different synthetic and system biology and analysis tools for the manipulation of yeast to boost yeast-based technology, broadening downstream products is also indispensable. For decades, demand has been rising for various commodities involving health, food, fuel, etc. Facing the shortage of market supply, more and more products are attempting to be produced using biotechnology due to the advantages of being fast, environmentally friendly, and low cost. 

Advanced yeast cell factories have been used for the production of natural products. Lycopene, a natural colorant with antioxidant properties, has been used as a common food additive [13]. Although *B. trispora* [14] and *E. coli* [15] have been engineered for lycopene production, the potential food safety issues could not be ignored in these two microorganisms. In comparison, yeast *S. cerevisiae* with GRAS is more promising as the lycopene microbial cell factory. Zhang et al. employed the genome-editing tool SCRaMbLE to evolve yeast *S. cerevisiae* to be compatible with the heterologous lycopene biosynthesis pathway. The expression of the optimized lycopene biosynthesis pathway improved the lycopene by 129.5 fold compared to the parental strain [16]. In addition, a semi-synthetic yeast strain harboring a synthetic chromosome II was evolved through SCRaMbLE, generating a new genotype of hygromycin B resistance [17]. These approaches could not only accelerate the process of optimizing yeast genotype, but also demonstrate that yeast mechanisms have a substantial effect on yeast phenotype. In addition to lycopene, isoprenoids, another typical secondary natural product, are important ingredients for flavored fragrance industries, pharmaceutical products, and natural rubber [18,19]. To enable yeast *S. cerevisiae* to efficiently produce farnesyl diphosphate (FPP)-derived isoprenoids, a combinatorial metabolic engineering strategy was adopted by improving the availability of the upstream FPP combined with removing the competitive reactions in the downstream, which significantly increased the product yield [20]. 

In addition to different metabolic engineering and synthetic and system biology strategies applied to modify yeast phenotypes, cultivation media directly influence strain analysis and strain performance in industrial applications [21]. In addition, with the increasing emphasis on safe and environmental issues, hazardous chemicals should be reduced or avoided in culture media based on the REACH regulation (EuropeanChemicalsAgency. Understanding REACH. Available online: https://echa.europa.eu/regulations/reach/understanding-reach, accessed on 20 December 2022). Therefore, optimizing the culture medium or fermentation broth is an indispensable step for applying yeast in biomanufacturing. For example, Basal Salt Medium (BSM) is a popular culture media for high-cell-density cultivations of *K. phaffii*. However, this defined culture media contains the harmful ingredients of boric acid and cobalt. Unfortunately, only a little knowledge of cultivations without cobalt or boric acid can be found. Therefore, Pekarsky et al. studied the impact of the depletion of boric acid and cobalt on high-cell-density cultivation, cellular morphology, viability, and recombinant protein production with *K. phaffii*, which provides a reference for future optimization of yeast media [22]. 

What is more, the application of yeast biotechnology should meet the requirements of modern society. For example, the COVID-19 pandemic once again reminded people of the importance of vaccine development. However, low- and middle-income countries face the issues of the limited availability and affordability of vaccines. The yeast *Pichia pastoris*, with its robust recombinant protein system, is one of the most promising hosts for vaccine production, which is promising to help low- and middle-income countries overcome these barriers to vaccination [23]. Another example is the wine industry; compared with traditional wine, the innovation in yeast-based wine-brewing technology could potentially address the challenges of the wine industry and provide the produced wine with freshness, a healthy flavor, and fragrances [24]. 

Yeast is a fantastic host being used for the development of engineering biology tools and strategies. Yeasts’ versatility, robustness, and ease of culture make them a valuable tool for advanced biomanufacturing. With the rapid development of genome-editing tools such as SCRaMbLE, synthetic biology tools, and artificial intelligence-guided machine learning models, yeast-based biotechnology will create more miracles in the near future.
